# Therapeutic effect of Fu's subcutaneous needling for hemiplegic shoulder pain among stroke survivors: Study protocol for a randomized controlled trial

**DOI:** 10.1097/MD.0000000000015507

**Published:** 2019-05-13

**Authors:** Tong Liu, Xi Wen, Weichuan Kuang, Xiaoyin Wang, Ye Jiang, Xiaojia Qiu, Yao Zeng, Guitao Zhang, Jiani Yu, Yue Liu

**Affiliations:** a Department of Acupuncture and Rehabilitation, GuangDong Second Hospital of Traditional Chinese Medicine; b Department of Acupuncture and Rehabilitation, Guangzhou University of Chinese Medicine; c Department of Rehabilitation Medicine, GuangDong Provincial Hospital of Traditional Chinese Medicine, Guangzhou, China.

**Keywords:** constant, Fu's subcutaneous needling, Fugl–Meyer, MPQ-SF, numerical rating scale, quality of life, range of motion, shoulder pain, stroke, usual care

## Abstract

**Background:**

Hemiplegic shoulder pain (HSP) is a frequent complication after stroke and limits patients’ physical functioning of the affected arm, thus compromising their quality of life. Fu's subcutaneous needling (FSN) has been widely applied in the treatment of pain diseases in China; however, its efficacy and safety for HSP remain to be elucidated. We therefore conducted a randomized, controlled trial to summarize the current evidence on the effects of FSN on the recovery outcomes of stroke survivors with HSP.

**Methods:**

Here, we conduct a study design and protocol of a randomized, blinded, controlled study to evaluate the efficacy and safety of FSN in patients with HSP. A total of 60 patients with numerical rating scale (NRS) score above 1 will be recruited in the trial and randomized into FSN group or usual care (UC) group. Patients in the FSN group will receive FSN treatment combined with UC treatment while patients in the UC group will receive UC treatment alone for 4 weeks. The primary outcomes are changes of NRS at baseline, after the 1st treatment, after the final treatment and 4 weeks after the final treatment. Secondary measurements will be changes of Fugl–Meyer score, constant score, MPQ-SF score, quality of life score, and range of motion at baseline, after the final treatment, and 4 weeks after the final treatment. The safety will also be assessed by monitoring the incidence of adverse events and changes in vital signs during the study.

**Discussion:**

Results from this trial will significantly support the application of FSN in the recovery of patients with HSP. If found to be effective and safe, FSN will be a valuable complementary option for patients with HSP.

**Trial registration:**

Chinese Clinical Trial Registry: ChiCTR1900021644 (registered on March 2, 2019).

## 1 Introduction

Hemiplegic shoulder pain (HSP) is a common and disabling complication among stroke survivors, with the rate of occurrence ranges from 16% to 84%.^[1–3]^ HSP may negatively affects patients’ range of motion (ROM) and motor functioning, thus withdraw rehabilitation programs and increase duration of hospitalization.^[4]^ Majority of factors may contribute to the occurrence of HSP, such as weakness of the rotator cuff muscles, subluxation, tendon inflammation, and shoulder–hand syndrome.

Accordingly, numerous treatment methods have been suggested, including percutaneous neuromuscular electrical stimulation (NMES), stretching exercises, pharmacologic therapy, shoulder positioning, strapping and taping, intraarticular corticosteroid injections, and so on. Nevertheless, inconclusive evidence was shown for the effectiveness of shoulder taping in reducing HSP.^[5–7]^ Manual techniques such as stretching, positioning, ROM functioning, or massage likely promote comfort only due to the lack of empirical evidence for their effects. Although corticosteroid injections may give satisfactory results, potential side effects include postinjection flare and tendon rupture.^[8]^ Percutaneous NMES requires invasive procedures to implant electrodes and poses the risk of electrode-related infections, which makes clinical implementation difficult.^[9,10]^

Acupuncture is an important component of traditional Chinese medicine and has been widely used in various diseases during the past 1000s of years in China. Many trials had explored the effect and safety of acupuncture for treating HSP,^[11–15]^ and a recent systematic review also concluded that conventional and electroacupuncture could be effective for management of HSP after stroke; however, very high potential for bias should be considered.^[16]^ Fu's subcutaneous needling (FSN) is a new acupuncture therapy, which mainly acts in the subcutaneous layer, like superficial acupuncture documented in “Huangdi Neijing” and developed from Ashi point therapy and wrist-ankle acupuncture therapy.^[17]^ Evidence has indicated that FSN could rapidly relieve the pain and obviously improve the spasm of soft tissues in treating the soft tissues injuries in clinical practice.^[18]^ Some studies had investigated the effect of FSN for HSP in China^[19,20]^; however, most of the trials are of low quality and well-designed randomized controlled clinical trials are still needed.

Thus, we present a study design and protocol of a randomized, blinded, controlled study to assess the efficacy and safety of FSN for treating HSP among stroke survivors. The objective of this study was to determine if FSN could strengthen the effect of usual care (UC) alone. The results of this study are expected to establish an optimal procedure for HSP.

## 2 Methods

### 2.1 Objective

In this study, the efficacy and safety of FSN combined with UC for HSP versus UC alone will be evaluated.

### 2.2 Trial design and setting

**Figure 1:**
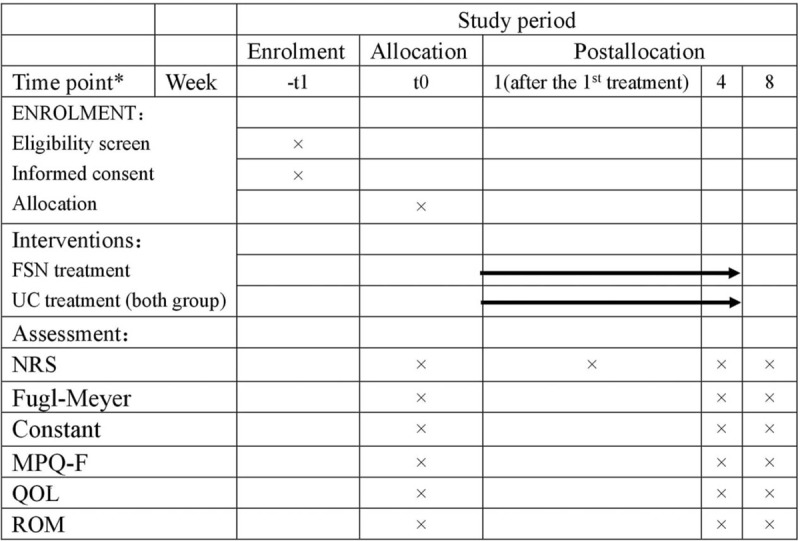
Schedule of enrollment, interventions, and assessments.

This study is a single-center, randomized controlled, assessor-blinded clinical trial which was devised following the Consolidated Standards of Reporting Trials (CONSORT) Statement recommendations^[21]^ and STRICTA.^[22]^ The total study period for this trial is 8 weeks (Fig. 1), including 4-week treatment phase and 4-week follow-up phase.

**Figure 2:**
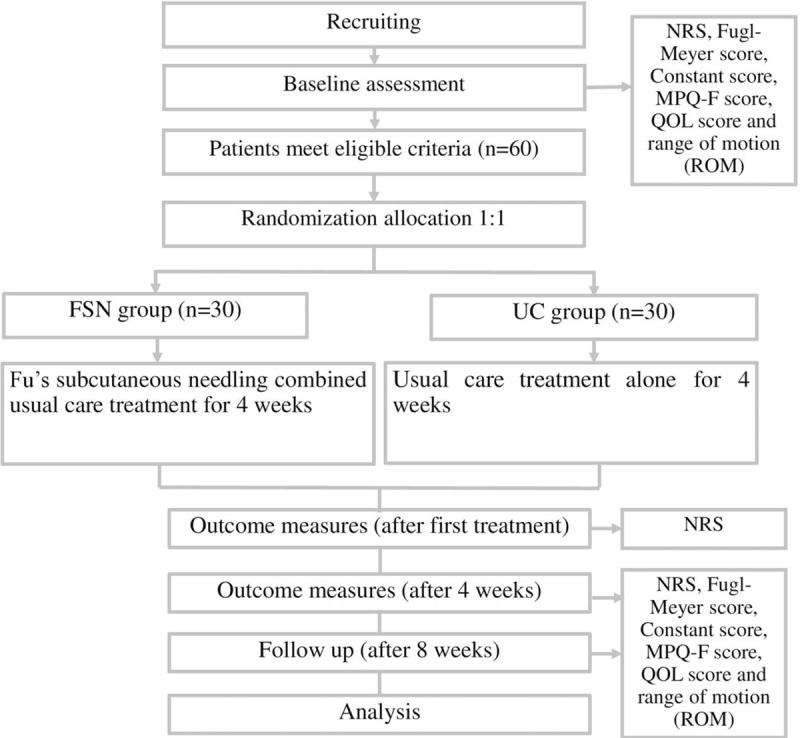
Flowchart of the study procedure.

The study will commence on June 1, 2019, and will be completed by December 31, 2020, at GuangDong Second Hospital of Traditional Chinese Medicine. Sixty patients who meet the eligibility criteria and sign an informed consent form will be randomly divided into 2 groups to receive either FSN combined with UC or UC treatment alone in a 1:1 ratio. The flowchart of the trial is shown in Figure 2.

## 3 Participants

### 3.1 Recruitment strategies

Inpatients at acupuncture departments in GuangDong Second Hospital of Traditional Chinese Medicine will be recruited mainly in this RCT. Additionally, posters will be put in the hospitals and a Chinese multipurpose social media named WeChat will be applied to recruit. Brief descriptions of eligible criteria, the free acupuncture treatments and the possible risks of the trial will be marked and all patients have the right to participate or drop out at any time, and will be required to sign the informed consent before the trial begin.

### 3.2 Eligibility criteria

The inclusion criteria will be as follows: HPS induced by stroke; Duration ranges from 2 weeks to 6 months at time of recruitment; age ranges from 20 to 80; intensity of HSP evaluated by numerical rating scale (NRS) was above 1; adequate communication ability and intact cognitive function (Mini-Mental Status Examination scores ≥24 points); and signed informed consent.

The exclusion criteria will be as follows: HPS not induced by stroke; combination of heart, liver, or kidney failure endanger the safety of life at any time; younger than 20 years or older than 80 years; those who do not receive acupuncture therapy; women with pregnancy and lactation; participate in other studies; aphasia, and severe cognitive deficits.

### 3.3 Randomization and allocation

Random numbers will be generated by the random number generator in the SPSS statistical software package (Version 20.0, IBM SPSS Statistics, IBM Corp, Somers, NY). Another specified researcher who is not involved in the study was responsible for it.

The allocation of participants will be sealed in a sequentially numbered opaque envelope. If the participant meets the inclusion criteria and signs informed consent, the above specified researcher will sequentially give the sealed random number envelope to the physician, who will open the envelope and allocate the participant to either FSN or UC group according to the random number.

### 3.4 Blinding

A single-blinded method will be used. While the participants and practitioners cannot be blinded, we blinded the outcome assessors, data manager, and statistics analyzer. The assessor will be instructed not to communicate with participants about the possibility of their treatment. James et al's blinding index will be evaluated after the completion of the study to evaluate the success of blinding.^[23]^

### 3.5 Interventions

In the 4-week treatment phase, participants in both groups will receive UC treatment, and those allocated to the intervention group will also receive FSN treatment.

### 3.6 UC treatment in both groups

Both groups underwent identical conventional rehabilitation programs including physical therapy and occupational therapy sessions, each lasting 60 minutes per day for 5 consecutive days per week. Traditional acupuncture therapy will be administered according to individual needs. Details of UC used during the trial will be recorded, noting any change of UC and the reasons.

### 3.7 FSN add-on treatment in the intervention group

**Figure 3:**
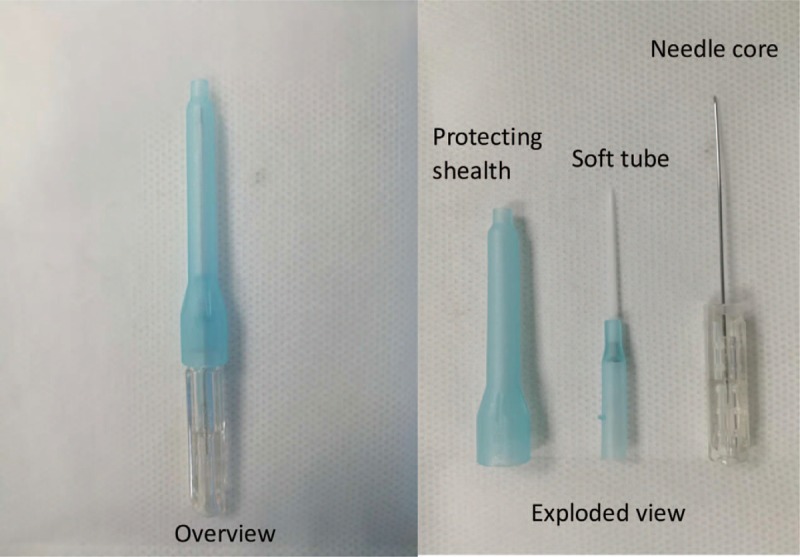
An overview and exploded view of the Fu's subcutaneous needling.

Patients will also be asked to take a lateral position with the affected limbs upside. For each patient, the positive reaction points (most significant tenderness points) around the affected shoulder will be identified and marked. Insertion points will be located 7 to 8 cm above or inferior to the points. After disinfection with 75% ethanol of each insertion point, a No. 6 disposable FSN needle (Fig. 3) was quickly inserted through the skin and into the subcutaneous layer. It was important to keep the needle tip aligned to the positive reaction point and just touched the muscular layer. Then draw a little back and push the needle forward toward the positive reaction point until the whole soft tube was beneath the skin. The steel needle was then drawed back 3 mm to avoid injuring blood vessels or other tissues during the following procedure. The needles were moved smoothly and rhythmically from one side to another horizontally 200 times in 2 minutes. Meanwhile, we will ask the patient to exercise the involved joints. If it did not make trouble with the movement, the steel needle will be pulled out while the soft tube of the needle remained under the skin for 8 to 24 hours.^[24]^ FSN will be conducted once daily, 5 times a week and lasted for 4 weeks.

### 3.8 Discontinuing interventions

The trial will be ceased if one of the following conditions appeared: a serious poststroke complication arises, or recurrent stroke or any other severe condition occurs leaving the patient in a critical condition.

### 3.9 Outcome measures

The primary efficacy endpoint will be NRS at baseline, after the 1st treatment, after the final treatment and 4 weeks after the final treatment. The NRS is a segmented numeric version of the visual analog scale (VAS) in which a respondent selects a whole number (0–10 integers) that best reflects the intensity of pain. Thus, we select NRS as the primary measurement to assess the improvement of pain.

Secondary measurements will be as follows:

Fugl–Meyer score: The FMA score consists of 17 items, ranging from 0 to 34, with lower scores demonstrating poorer movement function. In our trial, we just assessed the upper extremity Fugl–Meyer which includes 10 items and ranging from 0 to 20 at baseline, after the final treatment and 4 weeks after the final treatment.

Constant score: A supplementary assessment of function of the shoulder joint will be assessed by the constant score scale at baseline, after the final treatment and 4 weeks after the final treatment.

Short-form of McGill pain questionnaire (MPQ-SF) score: The MPQ-SF is a multidescriptive measure of pain that consists of 22 pain questions. It reveals the nature of shoulder pain. It will be evaluated at baseline, after the final treatment and 4 weeks after the final treatment.

Quality of life (QOL) score: QOL will be evaluated using the Chinese version of EQ-5D-5L^[25]^ at baseline, after the final treatment and 4 weeks after the final treatment.

ROM: Pain-free passive range of motion (PROM) of the shoulder was designated as the ROM attained in the most painful position and was measured using a digital goniometer in 6 directions: flexion, extension, abduction, adduction, external rotation, and internal rotation. Five directions of the shoulder joint were measured (flexion, abduction, adduction, internal rotation, and external rotation), while participants were lying in a supine position. In addition, shoulder extension was measured in the side-lying position. PROM was also evaluated at baseline, after the final treatment and 4 weeks after the final treatment.

### 3.10 Safety assessment

Any adverse events, including hematoma, infection, dizziness, and apostasies will be evaluated during the whole treatment by the researcher. If any severe adverse events occurring, FSN intervention will be ceased immediately and the principal investigator will be informed to take proper actions. One month will be followed up after the trial in case of subsequent adverse events.

### 3.11 Data collection

All the study data will be recorded on the CRF and any corrections made to the CRF must be personally signed and dated by the person responsible. All data will then be entered into a predesigned, password-protected electronic data set by 2 independent investigators who are blinded to group allocation. Double checking of entered data will be performed by another researcher to ensure accuracy.

### 3.12 Sample size calculation

The trial is designed to determine the role of FSN for treating HSP among stroke survivors. Therefore, NRS change will be used as an evaluation index. According to our previous pilot study, the change of NRS before and after treatment in the treatment group was shown to be 3.40 ± 0.48 (n = 10) and that in control group was 3.10 ± 0.23 (n = 10). Sample size was estimated using the following formula: 
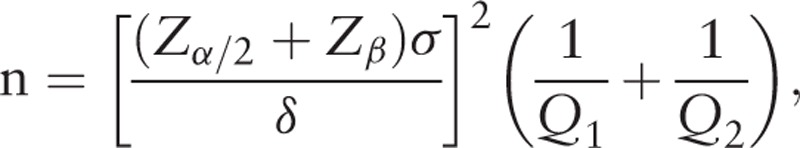


where n represents the number of samples required, n = n_1_ + n_2_, *Q*_1_ = n_1_/n, *Q*_2_ = n_2_/n with a significance level (*α* = 0.05) of a 2-sided 2-sample *t* test and 80% power to detect a difference between the 2 groups. Thus, a total sample size of 60 patients will be recruited allowing for 10% of attrition, with 30 in each.

### 3.13 Statistical analysis

The SPSS 20.0 (IBM SPSS Statistics, IBM Corp, Somers, NY) will be used to analyze the data. Quantitative data will be presented as mean ± standard deviation. NRS, Fugl–Meyer, Constant, MPQ-SF, QOL, and ROM scores will be conducted between the 2 groups by a superiority independent sample *t* test with a 95% confidence interval. The incidence of complications and adverse events will be compared by Chi-squared test. Any missing data will be replaced by the last measured value. For all analyses, *P* values of <.05 will be considered statistically significant.

### 3.14 Quality control

Before the trial, all the acupuncturists, nurses, and assessors will be trained strictly to guarantee homogeneity in the measurement data and ensure high-quality data results. The training content will include study protocol, diagnosis, inclusion, and exclusion criteria, recording method of CRF, FSN techniques, disposal of bleeding. All the study data will be recorded on the case report form. Dropouts and withdrawals from the study will be recorded in detail based on the intervention and follow-up periods. Data will be uploaded and verified by other 2 researchers who were not involved in the trial. This trial will be monitored by the Scientific Research Department of GuangDong Second Hospital of Traditional Chinese Medicine every 1 week.

### 3.15 Ethics and dissemination

We strictly follow the principles of the medical ethics of the Declaration of Helsinki^[26]^ with the approval of the Ethics and Research Committee of GuangDong Second Hospital of Traditional Chinese Medicine, China. If there are any protocol modifications, we will report to the Ethics and Research Committee for approval. All patients will be recruited from the Department of Acupuncture and Rehabilitation, GuangDong Second Hospital of Traditional Chinese Medicine. Informed consent will be obtained from all study participants. Participant information will be protected. All experimental data will be stored in a secure storage area with access limited to the researchers alone. We will disseminate the results of this study in meetings or publications when the trial is completed.

### 3.16 Patient and public involvement

This trial was designed to evaluate the add-on effects of FSN for HSP. In clinical practice, conventional acupuncture was widely used to treat complications of stroke, including HSP; however, the effect was always slow and not convinced for all patients. FSN may increase the effectiveness, hence significantly relieve pain and improve patients’ quality of life. The outcome measures used in this study were commonly used in clinical trials of HSP, and considered as important endpoints in our treatment. Patients with HSP after stroke in our clinical department were consulted prior to the trial design; in particular, the treatment frequency and duration of this trial were summarized from our clinical experience and patients’ feedback. The recruitment of patients will be carried out in our clinical department. However, those who were involved in the consultation related to the trial design will not be included as trial participants. Upon the completion of this trial, we will present the trial results in a peer-reviewed journal, or presented at international conferences. Once this manuscript is published, a brief summary of results using plain language will be sent to all trial participants. The burden of intervention will not be assessed by trial participants.

## 4 Discussion

This trial was designed to evaluate the effects and safety of FSN combined conventional acupuncture for the treatment of HSP. Evaluation of NRS, Fugl–Meyer, constant, MPQ-SF, QOL, ROM scores, as well as adverse events will be recorded and reported in a 4-week treatment and 4-week follow-up period. Moreover, this trial also involves incorporate appropriate allocation concealment and blinding of outcome assessors, data manager, statistics analyzer and will be reported in accordance with CONSORT 2010 and STRICTA 2010.^[21,22]^

Acupuncture has been practiced for more than 2500 years in China as an important therapeutic technique of traditional Chinese medicine. The effect of conventional acupuncture for HSP had been evaluated by several systematic reviews^[17,27,28]^; however, controversial conclusions were reached, which suggested that the effect of conventional acupuncture alone for HSP may be limited. FSN was invented by a acupuncturist in China named Fu-Zhonghua, and has been widely used in many clinical treatments.^[29–31]^ The needle-insertion layers of FSN is subcutaneous fascia, which has been a new research focus in recent years and considered to be closed related with pathologic mechanism of majority of diseases.^[32–34]^ According to FSN theory, the insertion points can be anywhere on the skin surrounding the painful spot and the distance between insertion point and pain spot is not fixed. In our trials, insertion points will be located 7 to 8 cm above or inferior to the pain spot to make the intervention repeatable. Moreover, patients should not feel numbness or aching during FSN intervention, which is similar to the characteristic of wrist-ankle acupuncture therapy.^[35]^ A recent study conducted by Jin et al^[36]^ had concluded that FSN combined with constraint-induced movement therapy could effectively improve spasticity and arm function in patients with stroke and spastic hemiparesis despite that it is just a case report. So we design this study to systematically observed the effect of FSN for HSP, to investigate if FSN could strengthen the clinical effect of UC treatment alone.

In our trial, strict quality control procedures have been applied to avoid bias, such as randomization and allocation concealment, assessor blinding and adequate sample size. However, methodologic limitations still exist. First, the trial is conducted in a single center. Thus, regional differences cannot be distinguished. Second, all the participants may have a higher expectation of acupuncture therapy because the trial was carried out in hospital of traditional Chinese medicine. Third, the therapists and the participants cannot be blinded in this study due to wide differences between FSN and UC treatment.

### 4.1 Trial status

This is protocol version 1.0, version date is November 11, 2018. Participants are not being recruited. Recruitment will begin on June 1, 2019. The trial is planned to be completed by December 31, 2020.

## Acknowledgment

The authors are thankful to Prof Nie Bin, GuangDong Second Hospital of Traditional Chinese Medicine, for scientific designment. They also thank all the patients and their caregivers who participated in this study.

## Author contributions

LT and XW: designed the study protocol and wrote the manuscript; WCK, YL, and XYW: acupuncture treatment; GTZ and YJ: outcome assessment; JNY: statistical analysis; XJQ: ethical approval; YZ and GTZ: follow-up. All authors approve the final version of the paper. The corresponding author has the sole responsibility of submission of the manuscript for publication.

**Conceptualization:** Tong Liu, Xi Wen, Yue Liu.

**Formal analysis:** Xiaojia Qiu, Jiani Yu.

**Funding acquisition:** Yue Liu.

**Methodology:** Ye Jiang, Xiaojia Qiu, Guitao Zhang.

**Project administration:** Weichuan Kuang, Xiaoyin Wang, Yao Zeng.

**Software:** Ye Jiang.

**Writing – original draft:** Tong Liu, Xi Wen.

**Writing – review & editing:** Jiani Yu.
